# Tutor’s Role in WhatsApp Learning Groups: A Quali-Quantitative Methodological Approach

**DOI:** 10.3389/fpsyg.2021.799456

**Published:** 2022-03-28

**Authors:** Susanna Annese, Francesca Amenduni, Vito Candido, Katherine Francis McLay, Maria Beatrice Ligorio

**Affiliations:** ^1^Department of Education, Psychology, Communication, University of Bari Aldo Moro, Bari, Italy; ^2^Research and Development, Sfuvet Swiss Federal University for Vocational Education and Training, Lugano, Switzerland; ^3^School of Education, The University of Queensland, Brisbane, QLD, Australia

**Keywords:** higher education, WhatsApp groups, tutoring, quali-quantitative method, thematic content analysis, social network analysis

## Abstract

In recent years, digital tools, such as WhatsApp, have been increasingly deployed to support group interaction and collaboration in higher education contexts. To understand contemporary, digitally-mediated collaborative dynamics – including the role played by tutors and the situated nature of group development – robust and innovative methodologies are needed. In this paper, we illustrate how integrating qualitative methods with quantitative tools used in qualitative ways makes it possible to trace how tutors adapt their style to support group development, which in turn triggers student development in a circular and responsive process. To make visible this contemporary phenomenon, we combine thematic content analysis – a qualitative tool – with a quantitative method: Social Network Analysis. Drawing on data generated by two WhatsApp learning groups (six students and four academic tutors) in research exploring the collaborative construction of boundary objects in a master’s level “E-learning Psychology” course, we suggest that our methodological approach has the potential to support interrogation of complex and dynamic digitally-mediated group interactions. Our results show the situational nature of an effective tutorship style through its complex adaptation to learners’ maturity, digital tools, and learning goals.

## Introduction

Studying group dynamics is challenging because of the intrinsic complexity associated with human interaction that involves constantly negotiating and renegotiating aims in situational context (Forsyth, 2018). Many methods attempt to analyze group dynamics, seeking to deploy theory and method in ways that are robust and account for the situated nature of groups. For instance, studying communication dynamics within an ecological context calls for an emic research perspective that directly involves social actors (Harris, 1976; Morey and Luthans, 1984). The emic perspective is also used to overcome the unhelpful dichotomy between qualitative and quantitative analysis. To this end, integrated approaches tend to be used; that is, qualitative methods are supplemented by quantitative tools used in a qualitative way. In this paper, we propose a method that combines, in a distinctive way, thematic content analysis – which is qualitative tool – with a quantitative method: Social Network Analysis.

Content analysis is one of the most frequently adopted approaches to interrogating group dynamics and is often used by social scientists to explore communication processes (Holsti, 1968). While the term “content analysis” covers a great variety of techniques, Krippendorff (1980) defines content analysis as, “a research technique for making replicable and valid inference from data to their context”. Traditional quantitative and qualitative approaches to content analysis differ in their interpretative procedures (Mayring, 2000, p. 21). The qualitative approach is also defined as “thematic content analysis” (Boyatzis, 1998) because qualitative data are encoded through specific thematic categories generated from and integrated with theory while scrutinizing the data, on the basis of what emerges.

One commonly used quantitative approach is Social Network Analysis (SNA), traditionally employed to explore the relational framework within groups (Wasserman and Faust, 1994; Scott, 1997) and to investigate both wide (Snyder and Kick, 1979) and restricted groups (Krackhardt, 1987; Galaskiewicz and Wasserman, 1989). SNA has also been used to investigate the relational framework of technology-mediated groups. For instance, Freeman and Freeman (1979) observed the interactions in a network of email exchanges among researchers, while Garton and his colleagues (Garton et al., 1997) used SNA to analyze collaborative online contexts. More recently, Martinez et al. (2003) had the idea to employ SNA in combination with content analysis to observe virtual groups engaged in collaborative learning settings.

But groups are living entities that change according to the affordances of the cultural and social context. Further, the tools used by groups to meet influence the nature of group dynamics. Nowadays, emails are no longer the optimal way to engage in group activities, and tools, such as WhatsApp, are becoming more popular. At the same time, research has become increasingly interested in more complex groups – those with more challenging goals and more specialized participation. Therefore, a more nuanced and complex methodology is needed; one that can account for the features of such groups as well as the digital context of group interaction.

To propose such a methodology, we first explain the nature of the groups we are interested in, then illustrate our methodological proposal. Finally, we demonstrate how to use the method by analyzing university groups that use WhatsApp to meet and to accomplish a complex professional task where two different types of tutors are involved.

## Inside the Groups: The Role of Tutors

Our research interest is in collaborative group work within formal learning contexts. Educational institutions are increasingly recognizing the value of group work, not only as a way of supporting collaboration (McCotter, 2001) but also as a means to blend different communities (Amenduni et al., 2021). Creating an intersection between different contexts implies the opening up of a “third” space that allows negotiation of meaning and the hybridity that makes available new cultural forms of dialogue (Akkerman and Bakker, 2011). In the present paper, we explore the intersection between universities and workplaces. We consider this intersection as crucial for students to engage in the transition to professional life by blending both theoretical and practical aspects, two components traditionally kept separate. Research indicates that to blend academic and professional experience and knowledge, a crucial role is played by both tutors and the digital tools deployed to support group processes (Bonk et al., 2005; Smith et al., 2009).

Some studies offer valuable insights into the impact of tutors’ roles and styles in blended approaches to higher education. For instance, Hytönen et al. (2016), in the context of an academic Apprenticeship Education Program in the field of energy efficiency, studied the extent to which interconnection between academic and workplace settings was considered as part of the tutoring process. They found that even with high-quality guidance from tutors in both contexts – university and workplace – the two settings remained separate. Tutors reported a few reasons why the interconnection did not happen; for instance, poor information about the practices or the general content of the training, and lack of joint meetings in which the course of action could have been collaboratively agreed upon. Participants also mentioned that they would have benefited from the academic and the workplace tutors engaging in more reciprocal interaction. This study indicates that stronger efforts from the organizing institutions are needed to support the interconnection between the tutors involved. This is particularly true when tutors belong to different contexts, such as academic and professional, which is the focus here.

Other studies show the functioning of the tutor in supporting small groups. Tutors engage students by identifying individual trajectories, monitoring each participant, and driving the whole group to participate in the collective activity (Chi, 1996; De Grave et al., 1999; Schmidt and Moust, 2000). Tutors answer students’ questions, improve their conceptual understanding (Graesser et al., 1995), enhance deep comprehension of the domain, and cultivate collaborative knowledge (Chi et al., 2001). This is possible because tutors help students compensate for their lack of knowledge through the expertise offered by tutors (Schmidt et al., 1993). Even groups with a low rate of productivity can take advantage of the presence of a tutor (Dolmans and Wolfhagen, 2005).

Several studies made clear that tutors’ style influences group dynamics and learning processes. For instance, Gerhardt-Szep et al. (2016), in the context of medical education, tested two different styles of tutorship: facilitative and non-facilitative. These styles produced different effects on the groups’ functioning and on knowledge construction. To identify and explore the effects, mixed methods were used, being an interesting example of mixed methodological approach for this research area. In fact, tutor effectiveness was assessed quantitatively by students’ questionnaires about knowledge before and after tutor intervention and an external observer’s rating of tutor performance. Qualitative analysis was based on a semi-structured interviews with tutors and focus group discussions with students. Quantitative findings showed significantly higher motivation and effectiveness with facilitative tutorship, while the non-facilitative style indicated a slightly greater improvement in students’ knowledge and understanding. Furthermore, external observers reported that the facilitative style implied significantly higher tutor involvement. Qualitative findings, however, revealed that a facilitative style was easier to take up for tutors but raised some concerns about students’ autonomy. During the focus group discussions, students regarded both styles as effective. However, they perceived the non-facilitative style as allowing greater independence in learning, whereas the facilitative style requires less learning “labor.” This research is particularly interesting because it shows how different methods can be combined to lead to complementary results.

Budé et al. (2009) compared – in the context of statistics education – tutors’ directive guidance with traditional guidance. The first approach involves a very active tutor who offers strategies to cope with problems and influences individual and group processes. The second approach involves tutors who limit their approach to providing general information about the course. The findings indicated that directive guidance was more positively received by students and also supported better learning outcomes.

These studies made clear how tutors should act when immersed in an academic context. However, more research is needed into how tutors should approach their role when supporting learning that crosses boundaries between two contexts, such as university and workplace. Situational Leadership Theory (Hersey and Blanchard, 1977) contends that the approach taken to leading style differs depending on the maturity levels of groups. In reviewing this theory, Hersey et al. (2008) better defined the group’s maturity levels by considering the developmental stage and performance readiness. However, the central assumption is that the task-relevant maturity level of the group is the main situational determinant of leading style. In fact, there is no best approach to leading style – both directive and supportive styles can be effective, depending on the involvement and expertise of the group.

Furthermore, Hersey et al. (2008) found at least four leading styles: telling, selling, participating, delegating. Telling is a directive style with some support and is appropriate for low maturity groups. Selling is a directive and supportive style, ideal for medium-low maturity groups. Participating is a supportive style with some directivity, recommended for medium-high maturity groups. Finally, delegating is a style without directivity or supportiveness, suitable for high maturity groups capable of independent operation.

Akkoyunlu and Soylu (2008) applied situational theory to blended learning contexts and conceptualized these four styles not merely as leading styles, but rather as learning styles or, more specifically, as guiding styles in learning dynamics. Meier (2016) maintained that leading roles in learning dynamics are nearly always situational because they fit students’ needs. He stated that situational guidance is a distinctive form of guidance that adjusts learning goals and digital tools to learners’ maturity level. So, the four guiding styles become four distinctive learning scenarios: if the learner is dependent, the guidance will be informative (telling) aimed at knowledge acquisition and the methodological tool could be instructions or tutorial. If the learner is engaged, guidance will be integrative (selling), aimed at supporting knowledge development, and the methods would deploy tools with moderate interactivity. If the learner is involved, guidance should be active (participating) to support knowledge practice, and tools can be fully interactive. If the learner is independent, guidance will be outsourced to students themselves (delegating) for autonomous knowledge construction, and methods will involve highly collaborative tools.

In our research, we build on these suggestions in understanding tutor action. Our focus is on academic tutors, examining how they guide learning groups through a blended context, within a digital environment created by WhatsApp.

## Why Use WhatsApp?

One of the crucial functions of technology is supporting users from different contexts to meet online and form new communities through hybridization and boundary crossing (Skeels and Grudin, 2009; Brooks, 2010). Various technologies have been interrogated from this perspective; among them, mobile technology has received substantial scholarly attention (Pimmer, 2016; Jiang and Edirisingha, 2019) because of its capacity to enable fluid movement between academic and professional contexts (Flynn et al., 2016).

Instant messaging – such as WhatsApp – is very popular among users of any age. WhatsApp has a great potential to enhance learning as it promotes participation, active study (Scornavacca et al., 2009; Cifuentes and Lents, 2010) and motivation to take part in academic assignments (Bere, 2013). It also removes social barriers between teachers and students by cultivating a sense of belonging to a community (Doering et al., 2008). Unlike forums, instant messaging makes students’ identity visible, allowing for personalized support (Hrastinski et al., 2014).

In educational contexts, WhatsApp provides flexibility as it can be used without time and space constraints, it is portable and accessible and makes it easy to share multimedia material (i.e., photos and videos). Furthermore, there is evidence that WhatsApp can enhance student learning outcomes, and that students perceive WhatsApp as valuable for learning and would like to use it more often (Cetinkaya, 2017).

Bouhnik and Deshen (2014) suggest that WhatsApp offers four key affordances: fast and easy communication; a pleasant environment; promotion of dialogue and sharing; and a simple platform. In addition, students recognized many educational advantages, such as learning materials’ availability, lessons beyond timetable hours, the opportunity to work in depth with fellow students, and teachers’ almost unlimited availability.

Looking more specifically at how WhatsApp is used when tutors are involved, Raiman et al. (2017) investigated six groups of medical students on clinical deployment monitored by tutors. The six instant messaging chats were examined through a thematic analysis whereas individual students were interviewed. The results of chats’ analyses highlighted the presence of three different types of processes: organizational, educational, and social. These processes allowed students to constantly arrange their work because they could always clarify learning goals or task details with their tutors. In the interviews with students, support for learning, ease of use, and availability of recorded discussions emerged as relevant features of the digital tool. In fact, interaction between students and tutors continuously occurred and most chat was produced by the students, who felt involved and at ease within the groups and with their tutors due to the hierarchical flattening. Furthermore, the availability of recorded discussions – as it happens in other asynchronous communication environments, such as web forums – allowed tutors to reflect on student participation and on the effectiveness of their own tutoring style.

Hrastinski et al. (2014) described informal coaching through WhatsApp groups with tutors (university students) training secondary education students in mathematics. Students stated that to learn effectively, tutors needed to know students’ competence level so coaching could be adjusted in response. This statement makes evident the situational determinant of tutorship style (as previously explained) and the focus on tutor’s instrumental activities that guide students to perform the task. Moreover, to accomplish learning goals, tutors had to establish personal relationships with each student as well as collaborative relationships among the group. This highlights the importance of tutors’ relational activities matched with the instrumental activities – the two functions of tutorship – and the tools’ affordances.

The positive evidence for using WhatsApp (So, 2016; Klein et al., 2018; Guo et al., 2020) can be extended also to boundary crossing practices (Coleman and O’Connor, 2019; Pimmer et al., 2019). The potentialities of instant messaging are clear; however, online group dynamics activated by tutors are still underexplored. In our research, we attempt to develop a method that can explore which group dynamics are activated when tutors support students in crossing boundaries between academic and professional contexts. To explore this phenomenon, we suggest that a purposely developed quali-quantitative method is needed. To test the method, we investigated two small group interactions within the WhatsApp environment.

## The Method

When blending different contexts – such as university and workplace – specific social interaction patterns may occur. This requires suitable methodological tools. In previous research (Ligorio et al., 2008; Annese et al., 2010; Annese and Traetta, 2011), we developed a distinctive approach using a quantitative method in a qualitative way. This integration produced a blended methodology, a quantified qualitative approach where qualitative content analysis was combined with SNA in different ways according to different research aims.

The method we propose integrates qualitative content analysis and SNA in three different combinations. In the first combination, we used SNA in traditional way: nodes signified participants and links signified interactions. Participants were identified, even for the problematic detection of the receivers in the group messages of digital contexts (Manca et al., 2009), by qualitative content analysis and represented by SNA as participation networks of blended learning communities (Annese and Traetta, 2012a). In the second combination we used SNA in a different way: nodes represented identity positionings of single participants and links were dialogical relationships among these identity positions. Positionings were identified through thematic content analysis and represented as positioning networks by an original version of SNA called PNA, Positioning Network Analysis (Annese and Traetta, 2012b). In the present paper, we propose a third and distinctive combination, that we employed for the first time in the research we report here. SNA is used in a similar but characteristic way, compared to Network Text Analysis where networks’ nodes are entities connected by the distribution of words or concepts (Popping and Roberts, 1997; Hunter and Sing, 2015; Mazzoni et al., 2015; Ravaglia et al., 2016; Celardo and Everett, 2020). In our research, networks’ nodes are co-occurrent categories resulting from thematic content analysis, and their links correspond to the connections among different co-occurrences, represented by SNA as category networks helpful to systematize internal and external links of co-occurrences in an inclusive graph. This enables exploration of social interactions and communication dynamics. We learned from Situational Leadership Theory (Hersey and Blanchard, 1977) that tutors adapt their interactional style according to the group’s needs and to environmental affordances. Our methodology supports the understanding of how this adaptation occurs by combining a qualitative tool – thematic content analysis – with a quantitative method: Social Network Analysis.

To demonstrate the robustness of this method, we tested it on small groups comprised of university students tutored by both academic tutors and tutors appointed by companies, but our research attention was for the peculiar interaction between academic tutors and university students. These groups communicated in a WhatsApp environment with the aim of building objects meant to cross-boundaries between two contexts – university and workplaces. The next paragraph will give details about the research context.

## The Research Context

The context under consideration is a blended course in “E-learning Psychology” at the University of Bari (Italy), where e-learning companies were invited to offer students the possibility to be engaged with professional activities. The theoretical framework supporting this aim is the Trialogical approach (Paavola et al., 2011), which suggests that collaboratively building shared boundary objects can support participants to transition from academic to professional contexts.

The course structure involves two modules: the first module covers the curricular content, while the second is focused on activities designed and performed in collaboration with companies. Here our focus is on the second module.

Academic tutors comprised volunteer students from previous course editions, who were interested in extending their e-learning expertise. They fulfilled this role as part of their internship or to collect data for their master’s thesis. The academic tutors were purposely trained, and their task was to act as intermediators between students, teacher, and company tutors. Academic tutors were expected to provide three kinds of monitoring: organizational (e.g., helping to meet deadlines), educational (e.g., supporting deeper reflection on the educational materials or eliciting connections among concepts), and technological (e.g., providing suggestions for specific demands about technology tools). Company tutors were employees or senior company representatives, and their task was to provide a business-oriented perspective and to guide the students in building the assigned boundary object. Beyond these general instructions, tutors were free to adopt the leading style they felt was most appropriate for the group.

The two companies involved were GruppoPragma (G) and Lattanzio (L), both active in the field of e-learning and interested in contributing to the course aim of supporting transition to professional contexts. In terms of the boundary objects produced, the first company asked students to produce ideas for the interface to be implemented in a MOOC^1^ training course for human resources managers. Students proposed the idea of “Personas” that prototyped the ideal manager of a big company. They described a few typical situations that such a manager could be involved in, and outlined some possible reactions. The second company proposed that students first become familiar with the concept of “microlearning” – an innovative learning approach particularly devoted to professionals and strongly based on portable technology – and subsequently, to develop a questionnaire to assess customer satisfaction.

Interactions between students and tutors occurred *via* WhatsApp and each group participated in two chats: one guided only by academic tutors and one guided by both academic and company tutors. Here, we focus on the chats guided only by academic tutors because they have more opportunity to interact with the students and therefore produce richer data.

The specific research questions guiding our analysis are:

1. Is our method suitable to identify and describe the adaptive dynamics of the academic tutor committed in guiding students in building cross-boundary objects?
2. How do the adaptative dynamics change the guiding style of academic tutors?

## Participants and Data

Twelve participants were involved: six students (average age 24), four academic tutors (average age 27), and two company tutors (average age 35) belonging to two different companies – Gruppopragma (G) and Lattanzio (L). For each company, two WhatsApp chats were available: one with the academic tutor only and another with both company and academic tutor.

As our aim was to investigate chats guided only by the academic tutor, our corpus of data comprises two chat logs including 740 posts (see Table 1). Chat-log transcriptions were divided into units of analysis corresponding to an argument chain which starts from a new discussion topic and ends when another topic is introduced (Strijbos et al., 2004, 2006). The criterion for segmentation of data in units of analysis is ecological, as it follows the natural organization of the collected dataset.

**Table 1:** Corpus of data.

Chat Logs	Posts	Units of analysis
G1 – only academic tutor	617	40
L1 – only academic tutor	123	15

The limited corpus of data is connected to the methodological approach, which requires an in-depth exploration through a quali-quantitative technique. In fact, as chat logs were spontaneously produced by groups in WhatsApp chats, they were systematically investigated through qualitative content analysis performed by the software *Atlas.ti*, combined with Social Network Analysis (SNA), applied with the software *Netminer*.

## Data Analysis

The data analysis required an iterative approach and involved three coders in total. At the beginning, two independent coders worked on 25% of data, later comparing divergent cases with a third coder until resolved. The following steps consisted of rounds of reading and coding, during which – at each round – the percentage of the data analyzed constantly increased until the whole corpus was coded and a final grid was produced (see Table 2). This is a non-mutually exclusive set of 15 hierarchically organized categories so that a unit could be coded with as many categories as were appropriate. The inter-coder reliability, calculated by *Atlas.ti*, was sufficient (Holsti index 88%) to consider the grid effective and replicable.

**Table 2:** Analysis’ grid.

Macro-Categories	Categories and Description
Decision-making	*Goal influence*: References to the goals when a decision has to be made
	*Task-structure influence*: References to the task structure when a decision has to be made
Role organization	*Students’ role*: References to the students’ role in the group organization; for instance, students may be in charge of finding more information or synthesizing the work
	*Relation with academic tutor*: References to the relationship with academic tutor
	*Relation with company tutor:* References to the relationship with company tutor
Interdependence	*Conflict:* Conflicts within or among groups
	*Collaboration:* Supportive and collaborative interactions with other students, whether in same group or not
	*Organization:* Interventions aimed at defining how to organize the work; for instance, establishing deadlines
	*Strengths/opportunities:* Comments about strengths and opportunities of the learning context
	*Challenges/weaknesses:* Comments about challenges and weaknesses of the learning context
Blended	References to the relationship between online and face-to-face dimensions
Psychosocial dynamics	Any other individual or collective process not included into the previous categories

Once the grid was stabilized, *Atlas.ti* first calculated the occurrences of categories (how often a category appears) and, later, the co-occurrences of categories (how often two categories occur together). Finally, SNA was used to schematically represent the links between co-occurrences.

## Results

To answer our research questions, we first report the results of occurrences and co-occurrences for thematic content analysis. Second, we treat co-occurrences through two SNA indices, and finally we explore in depth the network of the links within and between co-occurrences. Each step will be explained by quantitative and qualitative data in order to demonstrate the quali-quantitative nature of our methodological approach.

### Thematic Content Analysis

**Figure 1:**
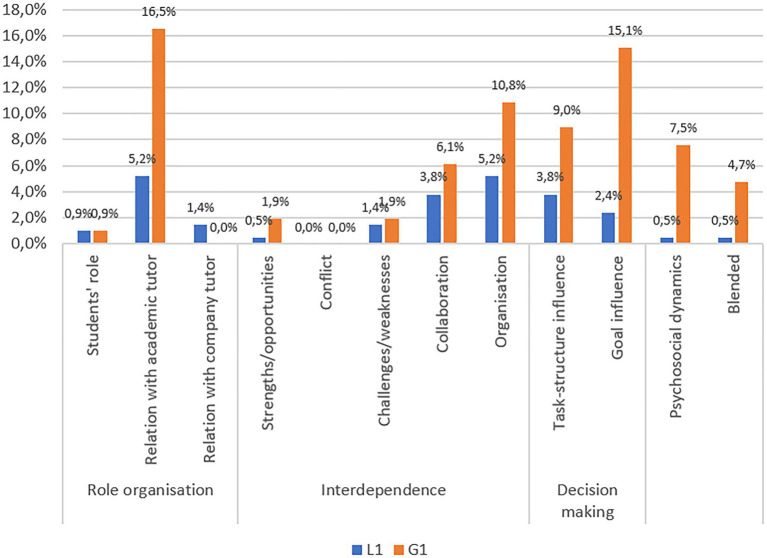
Occurrences in the two chats with only academic tutors (L1-G1).

Analysis of occurrences in the chats involving only academic tutors reveals that there are three macro-categories: Interdependence, Role organization, and Decision-making (see Figure 1). When considering the two different chats together (L1-G1), a specific composition of the three main macro-categories becomes clear: Interdependence centered on Organization; Role organization focused on the most recurring category Relation with academic tutor; and, finally, Decision-making activated by both Goal influence and Task-structure influence.

Interaction within these groups features strong Relation with the academic tutor, who manages Organization by orienting it toward both Goal Influence and Task Influence. In fact, students turn to the academic tutor for a range of requests, even questions that would be more appropriate to be addressed to the company tutor (see “Excerpt 1 – L1”).

#### Excerpt 1 – L1

Student 1: *Hi Academic tutor*^2^ *I have to ask you a clarification … When the company tutor spoke about three weeks’ time, was he referring to the work of the two sub-groups, right? Not to the whole work… Or am I wrong?*

The academic tutor also takes care of the whole organization of the interaction, which is strongly dependent on the goal to be achieved – building objects with companies. The tutor seems to be fully aware that this goal can be achieved only through the tasks students perform, urging them not to lose focus (see “Excerpt 2 – G1”).

#### Excerpt 2 – G1

Academic tutor: *Hi girls, I invite you, as soon as you can, to write on the forum what you are doing and fix intermediate deadlines of the various phases for designing and realizing the product.*

Student 1: *Hey Academic tutor, you are right, I am taking too long with this micro-design.*

In this case, the academic tutor brings the group’s attention to the need to make visible the micro-design method, previously agreed with the company tutor. The goal – the product to be delivered – does not only impact the group’s organizational dynamics but also the academic tutor’s role, who guides organizational strategies and the structure of micro-tasks. In general, analysis of occurrences indicates that Relation with the academic tutor is the driving force of the group.

This occurrence’s description requires further exploration through the analysis of co-occurrences. *Atlas.ti* performs this investigation through specific analysis operations that the researcher chooses according to the research aims. *Atlas.ti* reports the results of co-occurrence analysis through a c-coefficient calculated as follow: c = n12/(n1 + n2 – n12). This is a coefficient that indicates the strength of the relation between two categories, similar to a correlation coefficient (Armborst, 2017).

Co-occurrences in the chats managed by only academic tutors are numerous, with high frequencies and a large variety of categories (see Table 3). The most dominant is between Goal influence and Relation with academic tutor, but those between Goal influence and Organization, and Organization and Relation with academic tutor are also essential to feature the relationships within the group. We suggest that the boundary crossing object is the catalyst for these three specific co-occurrences.

**Table 3:** Co-occurrences in the two chats with only academic tutors (L1-G1).

	BL	COLL	CONF	ORG	PSYCHOS	TASK	GOAL	OPP	COMP_ TUT	ACAD_TUT	STUD	CHAL
BL	0.00											
COLL	0.23	0.00										
CONF	0.00	0.00	0.00									
ORG	0.25	0.38	0.00	0.00								
PSYCHOS	0.17	0.12	0.00	0.16	0.00							
TASK	0.27	0.33	0.00	0.39	0.16	0.00						
GOAL	0.26	0.35	0.00	0.61	0.23	0.39	0.00					
OPP	0.07	0.13	0.00	0.08	0.05	0.14	0.11	0.00				
COMP_TUT	0.00	0.04	0.00	0.06	0.00	0.07	0.03	0.14	0.00			
ACAD_TUT	0.24	0.40	0.00	0.57	0.26	0.43	0.66	0.09	0.04	0.00		
STUD	0.00	0.09	0.00	0.03	0.00	0.11	0.05	0.50	0.40	0.06	0.00	
CHAL	0.06	0.22	0.00	0.14	0.04	0.06	0.10	0.09	0.00	0.13	0.10	0.00

BL, blended; COLL, collaboration; CONF, conflict; ORG, organization; PSYCHOS, psychosocial dynamics; TASK, task-structure influence; GOAL, goal influence; OPP, strengths/opportunities; COMP_TUT, relation with company tutor; ACAD_TUT, relation with academic tutor; STUD, Students’ role; CHAL, challenges/weaknesses.

0,15 < C < 0,20, 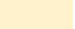; 0,21 < C < 0,25, 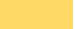; 0,26 < C < 0,35, 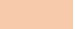; 0,36 < C < 0,45, 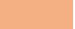; 0,46 < C < 0,55, 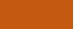; C > 0,55, 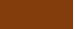

The co-occurrence between Students’ role and Strengths/opportunities is also interesting because it shows the improvement of these two features of group interaction through a mutual relationship.

Other relevant co-occurrences are between Relation with academic tutor and Task-structure influence; Relation with academic tutor and Collaboration; and Relation with company tutor and Students’ role. These co-occurrences are interesting because the role of the academic tutor is now related not only to the task’s structure but also to the collaborative dynamics. The co-occurrence between the role of the company tutor and that of the students highlights an enhancement of the reciprocal relationship.

Last, co-occurrences that can be underlined are those between Task-structure influence and Organization; Task-structure influence and Goal influence; and Organization and Collaboration. Far from being the last in terms of quantitative relevance, these co-occurrences show once again the centrality of the task and of the organization, connected to the collaborative dynamic.

These co-occurrences indicate the tutor’s focus on both practical (goal, task, and organization) and socioemotional (students’ role, their relationship with company tutors, collaboration) features of the group. The collective goal – building a boundary object – also impacts the tutor’s dual function: instrumental, meant to manage the organizational dimension; and relational, concerning the socioemotional dimension.

### Social Network Analysis

The academic tutor plays a very distinctive role and triggers specific group dynamics, clearly emerging when co-occurrence analysis is combined with the SNA and particularly with two indices: neighbor analysis and centrality analysis. To better understand this network of categories, we imported the co-occurrence matrix into *Netminer*, to generate an overall representation of both internal links (within each co-occurrence) and external links (among the various co-occurrences). Neighbor analysis shows the density of links, whereas centrality analysis shows central co-occurrences in the overall representation.

**Figure 2:**
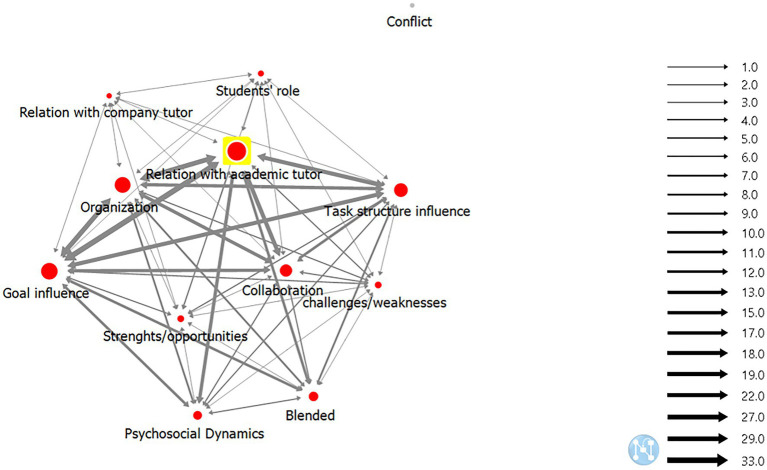
Neighbour analysis of co-occurrences in the chats with only academic tutors (L1-G1).

In neighbor analysis, the final graph of the links between categories (see Figure 2) makes visible two factors: (i) the centrality of the academic tutor and (ii) group endeavor to accomplish the goal and perform the task. The whole interaction profile of the chat, from the organization to the collaborative relationships, is based upon these two factors.

In the neighbor analysis, a cohesive representation of the internal and external links of co-occurrences emerges with one exception: the Conflict category, which is an isolated node with no link to any of the other categories. This category was never assigned to any interaction.

Crucial occurrences for the cohesion of the category network are, from largest to smallest: (i) Relation with academic tutor, the driving force for the whole network; (ii) Goal influence and Organization, central nodes for the network; (iii) Task-structure influence, important category for the network; and (iv) Collaboration, the last of central nodes. The neighbor analysis also makes it possible to track down the strength of co-occurrences in the category network through the links between nodes: the more the co-occurrence is quantitatively significant, the more the link is thick in the graphical representation (see Figure 2).

The density of links in graphical representation effectively illustrates the configuration of co-occurrences that emerged during the thematic content analysis. The densest link (33.0) refers to the co-occurrence between Relation with academic tutor and Goal influence, which means that the collective goal shapes the strategies and activities of the academic tutor. Other essential links for the cohesion of the category network are those between Goal influence and Organization (29.0), where the goal affects the organizational dynamics, and between Organization and Relation with academic tutor (29.0), indicating that the academic tutor monitors the whole group organization. Excerpt 3 is a good example of how the academic tutor takes charge of organizing the activities.

#### Excerpt 3 – G1

Academic tutor: *In my opinion, we first should ask for the material, so you can start analysing it.*

Academic tutor: *And as soon as you can, you should download the software.*

Student 2: *Sure, we need the material to get a better idea of what to do.*

The academic tutor chooses what organizational strategy is the most appropriate (download the software) and breaks it into specific times and tasks, showing a focus on the instrumental dimension in the guiding role. In fact, a dense link in the category network is that of the co-occurrence between tasks and tutors, namely between Task-structure influence and Relation with academic tutor (27.0). Even when the task concerns the academic tutor herself, she involves the students in checking on her task (see “Excerpt 4 – L1”).

#### Excerpt 4 – L1

Academic tutor: *Is there any information I omitted? Anything you would like me to specify?*

Student 1: *No Academic tutor, it’s ok for me.*

Student 1: *At the end it has been a work general enough.*

In this excerpt, the tutor refers to a document she wrote to summarize the work done by the group to inform the company tutor and she actively involves the students in the collaborative structuring of this report. In this regard, the co-occurrence between Collaboration and Relation with academic tutor is another dense link (27.0) in the category network. The tasks, the instrumental dimension of the tutorship, and the group organization become an opportunity for the tutor to trigger a collaborative strategy.

From this emerges a tutor style that may appear directive as it is strongly centered on the Task-structure, with dense co-occurrence links with both Goal influence (22.0) and Organization (22.0). The instrumental function of tutorship and the goal-oriented interaction push the group to ask for such directivity, and the tutor leverages this to activate collaborative dynamic. This is probably why Collaboration has dense co-occurrence links with Goal influence (19.0) and Organization (19.0). Through goal and organization, the tutor attempts to improve the collaborative dynamics, activating the relational function of tutorship role. In this function, the tutor shifts from a directive to a supportive style and, at the same time, pushes the group to develop collaboration and autonomy.

**Figure 3:**
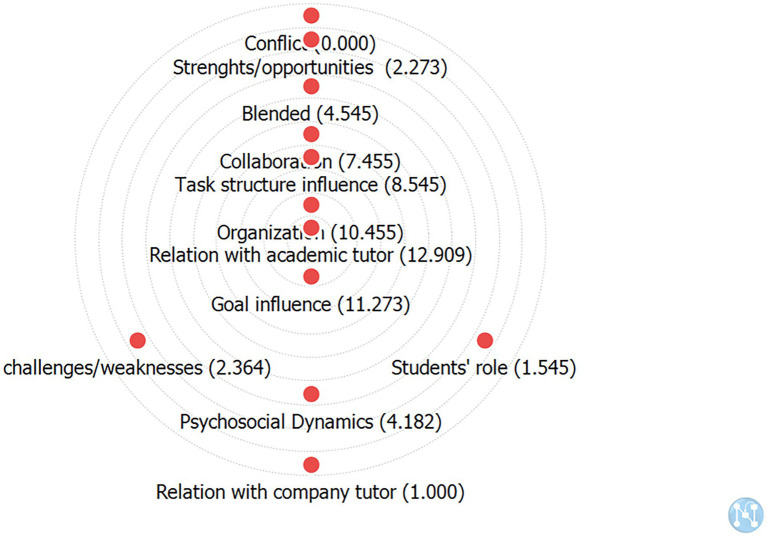
Centrality analysis of co-occurrences in the chats with only academic tutors (L1-G1).

The results from the neighbor analysis are confirmed by centrality analysis (see Figure 3) which reports the averages of in-degree and out-degree centrality for different co-occurrences, highlighting the most central categories in the network.

Relation with academic tutor (12.909) is the most crucial, followed by Goal influence (11.273), and Organization (10.455). In contrast, Task-structure influence (8.545) and Collaboration (7.455) are the last of the central categories. This tutor produces a directive style in his instrumental function, leading the group toward positive interdependence and collaborative relationships that allow the group and the individuals to evolve. This evolution allows the tutor to focus more on the relational function and to move toward a supportive style.

Neighbor and centrality analysis turned out to be important in answering our research questions: they showed the academic tutor’s style and its impact on the interactive profile of the group (second research question), but they also revealed how the dual function – instrumental and relational – of this role adapts to the degree of evolution of the group (first research question). The strength of the links between the co-occurrences and their centrality have indicated a clear connection between the tutorship style and the situational factors: the academic tutor shifts style back and forth from directive to supportive, according to the changes in the group maturation and the flow of the interactive situation.

### Co-occurrences in Depth

To better illustrate the specificities of tutor style and its adaptivity to situational factors, we now explore in depth the co-occurrences found in group dynamics of chats managed by academic tutors only (see Table 3). The most relevant is that between Goal influence and Relation with academic tutor because the goal shapes the tutor’s role. The following excerpt (5) provides a good example.

#### Excerpt 5 – G1

Academic tutor: [to one student] *I understand your state of confusion, I was confused, too hahaha.*

Student 1: *I was just about to say that.*

Academic tutor: *Honestly, I was expecting a short tutorial for the software.*

Prior this exchange, students and the tutors met in a Skype call to discuss the object to be created. The academic tutors could sense the students’ concerns. The academic tutors adopted two different strategies to manage students’ anxiety: using irony to play down the stress and let students feel free to express their doubts and sense of confusion and offering to support students by obtaining further material and advising students to download the software. These two strategies clarify how the goal influences the dual function of tutorship: organizing the executive aspects of the goal to be achieved and managing the socioemotional aspects of relationships. Therefore, both the instrumental dimension and the relational dimension are evident.

Two other co-occurrences are also relevant: Goal influence and Organization, Organization and Relation with the academic tutor. In these co-occurrences, once again the boundary crossing object becomes the catalyst, possibly because of the organizational dynamic created by the academic tutor. Therefore, the goal not only shapes the academic tutor role, but also the organizational dynamics of the group, as made clear by excerpt 6.

#### Excerpt 6 – G1

Academic tutor 2: *Anyway girls, consider the free version of the software, which I think lasts a month.*

Student 2: *I will try to do as much as possible by Friday.*

Academic tutor 1: *you will be delighted to celebrate your birthday in this way.*

Academic tutor 2: *Not all of you should download the software. Save a PC where to download it later.*

The goal to build the cross-boundary object is at the center of the group interaction and the academic tutor guides the organizational strategy, carrying out the instrumental dimension of the role. In this instrumental dimension, the co-occurrence of Students’ roles and Strengths/opportunities is relevant because it represents enhancement of these two aspects, operationalized by the academic tutor (see “Excerpt 7 – L1”).

#### Excerpt 7 – L1

Academic tutor: *Hello @ Company tutor, as expected I proceed with a report of the activities carried out by my group. At the moment, the students have paid attention to how the materials are presented, annotating when they were in video format, slides, texts. […] For this reason I encouraged them to look at the materials and wonder what other knowledge could be useful to integrate into this course without necessarily binding to the thematic organization proposed.*

In this post, the academic tutor values the active role of the students and indicates how they can further enrich their work in progress. The tutor is adapting the instrumental function of the role to the situational characteristics of the group because, by enhancing the students’ role, tutor is intertwining the relational dimension with the instrumental one.

The tutor’s guidance is characterized by the instrumental strategy and the co-occurrence between Relation with academic tutor and Task-structure influence foregrounds how the collective objective is structured through operational tasks. Two other interesting co-occurrences – Relation with company tutor and Students’ role, Relation with academic tutor and Collaboration – also show the tutor’s relational attention to collaborative dynamics. In fact, in the co-occurrence between Relation with company tutor and Students’ role, the tutor fosters a relationship (see Excerpt 8) between students and company tutor.

#### Excerpt 8 – L1

Student 2: Ah *Academic Tutor, I have to ask you a clarification … When company tutor spoke about three weeks’ time, was he referring to the work of the two sub-groups, right? Not the whole work.. Or am I wrong? Do you want me to ask to the company tutor in our group?*

Academic tutor: *Yes, of course you can ask the questions directly to him. I can tell you that our commitment with the company ends on January 22nd. So I think three weeks is the overall time.*

Academic tutor: *However, in general if you have any doubt you can ask the questions directly, the group also includes you so that you can speak directly!*

Here we see that, in the co-occurrence between Relation with academic tutor and Collaboration, the intertwining of the instrumental dimension and the relational dimension of the tutorship is oriented toward a collaborative strategy (see “Excerpt 9 – L1”).

#### Excerpt 9 – L1

Student 1: *So from now on we start to go in depth and to create connections in the material; this is fine with me.*

Academic tutor: *Ok when your groupmate gives us the ok, I’ll send it to the group.*

Student 2: *Yes, it’s okay.*

In this exchange, a student would like to start an activity and the academic tutor makes sure that the other students are involved to ensure the activity is collaborative. The instrumental dimension of tutorship, evident in the centrality of the Task-structure influence category in co-occurrences with Goal influence and Organization, is intertwined with the relational dimension, focusing attention on the collaborative dynamics. In fact, the last of the most interesting co-occurrences is between Organization and Collaboration (see “Excerpt 10 – G1”).

#### Excerpt 10 – G1

Student 1: *Try to access through this link (link to their object).*

Academic tutor: *We can visualize it but not modify the content.*

Student 1: [to the tutor] *I tried to add you as a collaborator but it tells that it is still sending the request.*

Academic tutor: *However, you can do it also in another way… We will discuss this tomorrow in class.*

The students are trying to share the link of their object with the academic tutor to allow her to see it and also to contribute collaboratively to changes. Because of the difficulties in sharing the link, the academic tutor proposes an organizational strategy: to postpone the solution to the following day during the face-to-face lesson.

In summary, when tutors interact with students who are engaged but not autonomous in their work, the tutor’s instrumental and directive style can lead the group to refine its skills. When students are already involved, the tutor’s relational and supportive style can lead the group to be more collaborative. It seems that the relational function of tutorship style pushes individual members toward greater involvement and the whole group toward collaborative work.

In other words, an adaptive tutor who shifts from directive to supportive style, not only guides task performance and improves students’ skills, but also increases student confidence and social effectiveness. A flexible tutorship style represents an adaptive response to the situation and to the evolving group and is a driving force behind group development.

## Discussion and Conclusion

In this paper, we propose a distinctive and robust method to identify and describe the flexibility tutors need when monitoring online group interactions in relation to challenging tasks involving boundary crossing between university and workplace. We developed this methodological approach to answer our two research questions: first, is our method suitable to identify and describe the adaptive dynamics of the academic tutor committed in guiding students in building cross-boundary objects? And second, how do the adaptive dynamics change the guiding style of academic tutors?

In relation to the first research question, we suggest this research indicates that our method makes it possible to identify and describe how tutors structured their interventions, how the groups responded to these interventions, and how the tutors subsequently adjust their styles. In short, our methodological approach effectively supports in-depth investigation of group dynamics in an online context. The combination of a qualitative tool and a quantitative method employed in a qualitative way highlights the relationship between group characteristics and tutor guidance. Therefore, we hope this method can be applied in future research in similar contexts. In fact, we consider this method useful when at least three conditions are present: (i) groups are asked to work on the boundaries between different contexts, such as university and professional life; (ii) complex and challenging tasks are proposed; and (iii) a digital environment is used – WhatsApp and many similar tools even more supportive in tracking down the thread evolutions, such as Slack or Trello – which allows all interactions to be recorded.

In relation to the second research question, we found that academic tutors are focused on the organizational dynamics necessary to accomplish the goal, and this produces a convergent group interaction with no conflicts. Tutor guidance is based on the instrumental dimension without neglecting the socioemotional aspects. The academic tutors’ objective seems to be group growth and, to this end, they shift their style from directive to supportive depending on the situation. Tutors move from a directive style (task-centered) of tutorship toward a supportive style (relationship-centered) according to the level of development and maturity of the group. When group interaction is centered upon the instrumental dimension, the academic tutor tends to be more directive than supportive. In contrast, when group interaction is centered upon the relational dimension, the academic tutor tends to be more supportive than directive. This adaptive tutorship style triggers student development, the group evolves in response, and tutors again adjust their style – in a circular influence process.

Our results confirm the relevance of the tutors in supporting online group work (Raiman et al., 2017) and in fostering students’ deep understanding (Graesser et al., 1995) by activating knowledge construction (Chi et al., 2001) through participation. Our results also align with research into the influence of tutorship style on group learning processes (Gerhardt-Szep et al., 2016), and with research that contends that effective tutorship style is situational and responsive; that is, tutor guidance adjusts depending on learning goals, the digital tools being deployed, and learner maturity (Hrastinski et al., 2014; Meier, 2016).

The relevance of the academic tutor prompts new research directions about the various features of this role in instant messaging learning groups; for example, the kind of students who benefit from directive or supportive tutorship. Additionally, future research could explore this role in diverse contexts as well as other complex higher education learning communities.

While our research foregrounds the relevance of the academic tutor role, we suggest it is also important to explore the influence of representatives from the blended context (such as company tutors, as in our research). Future research could explore how to better integrate academic and professional tutors, for example.

Finally, we are aware that a limitation of our work is that the dataset and the sample are both small. Nevertheless, we believe our work can contribute in defining sophisticated methods, aimed not only at offering a snapshot of a specific situation, but also able to track down flexible and changing situations, as it occurs in life and especially in an educational context, with complex objectives and fixed deadlines.

We would like to see our method evolve and be tested through further applications and also by triangulating data from different sources. A possible follow-up to this method could be an integrated analysis of data generated from tutor and/or student interviews or from researcher field notes developed through online and/or in-person observation. This could support a multi-dimensional and dynamic picture of complex contemporary phenomena. We believe our work can contribute to developing robust methodologies capable of capturing complex and dynamic digitally-mediated group interactions that are increasingly prevalent in educational and professional contexts.

## Data Availability Statement

The data analyzed in this study is subject to the following licenses/restrictions: dataset may contain sensitive-context information, so only a portion of data can be made available. Requests to access these datasets should be directed to susanna.annese@uniba.it.

## Ethics Statement

Ethical review and approval was not required for the study on human participants in accordance with the local legislation and institutional requirements. Written informed consent for participation was not required for this study in accordance with the national legislation and the institutional requirements.

## Author Contributions

SA – methodology, investigation, and writing-original draft. FA – data analysis and validation. VC – data analysis and software. KFM – english review, editing, and abstract. MBL – conceptualization, writing-paper finalization, and supervision. All authors contributed to the article and approved the submitted version.

## Funding

The authors thank the University Aldo Moro of Bari for funding the Open Access publication fees of this manuscript.

## Conflict of Interest

The authors declare that the research was conducted in the absence of any commercial or financial relationships that could be construed as a potential conflict of interest.

## Publisher’s Note

All claims expressed in this article are solely those of the authors and do not necessarily represent those of their affiliated organizations, or those of the publisher, the editors and the reviewers. Any product that may be evaluated in this article, or claim that may be made by its manufacturer, is not guaranteed or endorsed by the publisher.

## Footnotes

^1^Massive Open Online Course, in Italian: these are courses designed for distance learning involving a large number of users.

^2^To protect tutor’s identity and still make clear to whom students are talking, we adopted this expression.
